# A Semi-Analytical Model for the Heat Generation during Hybrid Metal Extrusion and Bonding (HYB)

**DOI:** 10.3390/ma14010170

**Published:** 2020-12-31

**Authors:** Francesco Leoni, Øystein Grong, Paolo Ferro, Filippo Berto

**Affiliations:** 1 Department of Mechanical and Industrial Engineering, Norwegian University of Science and Technology, Richard Birkelands vei 2b, 7491 Trondheim, Norway; professor.grong@gmail.com (Ø.G.); Filippo.berto@ntnu.no (F.B.); 2 HyBond AS, NAPIC Richard Birkelands vei 2b, 7491 Trondheim, Norway; 3 Department of Management and Engineering, University of Padova, Stradella S. Nicola, 2 I-36100 Vicenza, Italy; paolo.ferro@unipd.it

**Keywords:** aluminium butt welding, hybrid metal extrusion and bonding, frictional heating, heat flow modelling, thermal efficiency factor

## Abstract

Hybrid Metal Extrusion and Bonding (HYB) is a novel solid-state welding method for metals and alloys that utilises continuous extrusion as a technique to enable aluminium filler metal additions. In the present study, a new semi-analytical model for the heat generation during aluminium butt welding is presented. As a starting point, the classical Rosenthal thin plate solution for the pseudo-steady-state temperature distribution around a fully penetrating line source is invoked. Then, the associated heat generation is calculated by considering the individual contributions from the tip of the rotating pin, the pin shoulder, and the filler metal additions on the net power input. In a calibrated form, the model yields thermal efficiency factors that are in close agreement with those obtained from more sophisticated finite element analyses but with considerably less computational effort.

## 1. Introduction

The term solid-state joining covers a vast number of processes including diffusion welding, explosion welding, hot pressure welding, forge welding, ultrasonic welding, cold pressure welding, roll welding, friction stir welding, and conventional friction welding [1,2]. All these processes operate at temperatures below the melting point of the base materials to be joined. As a consequence, the welds produced will largely retain their microstructural integrity without forming a fusion zone and a wide heat-affected zone (HAZ) with degraded properties, which is the main problem with traditional fusion welding [3,4]. In addition, they allow the joining of materials that are known to be prone to hot cracking and pore formation, with no hazardous fume formation, radiation from an open arc, or oxidation losses of alloying elements [5,6,7,8,9]. Therefore, the benefits of solid-state joining are obvious and well documented.

**Figure 1:**
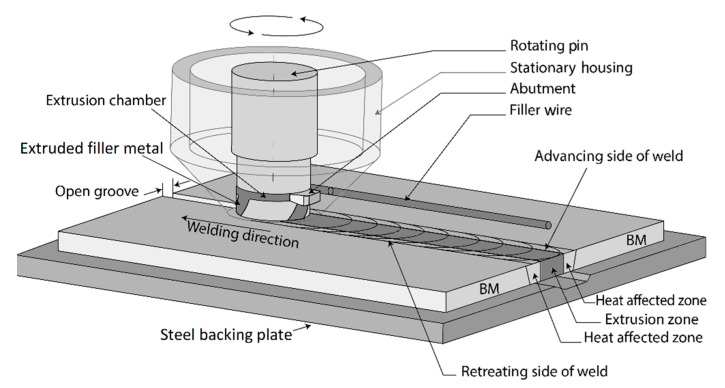
Schematic illustration of Hybrid Metal Extrusion and Bonding (HYB) PinPoint extruder in a butt-welding situation.

Recently, a new solid-state joining method for metals and alloys has been developed, known as the Hybrid Metal Extrusion and Bonding (HYB) process [10,11,12,13,14]. This method, which is based on the principles of continuous extrusion, allows joining to be performed using aluminium filler metal (FM) additions similar to that done in gas metal arc welding (GMAW) but without any melting involved. Figure 1 highlights the core parts forming the HYB PinPoint extruder. In the situation of butt-welding, the two plates to be joined are divided from each other by a fixed spacing so that an I-groove forms between them. During the welding operation, the PinPoint extruder shown in Figure 1 moves along the joint line at a constant travel speed, while the rotating pin with its moving dies is submerged between the plates. This enables the extrudate to flow downwards in the axial direction and mix with the base metal (BM) in the groove under the conditions of high pressure and severe plastic deformation. Metallic bonding between the FM and the BM occurs as a result of these two contributions along with oxide dispersion [10,11]. By taking into consideration the mass balance involved, the extrudate flow rate can be adjusted (by using the rotational speed of the drive spindle as the main process variable) in such a way that the groove between the plates is filled with solid aluminium in a continuous manner [10,11,12,13,14].

Since HYB is based on the principles of continuous extrusion, it will differ from traditional friction stir welding (FSW), although some similarities exist. Furthermore, because HYB is a new process, it has not yet been explored to the same extent as FSW, where different coupled thermomechanical models already exist in the scientific literature [15,16,17,18,19]. For example, Ulysse [16] has developed a 3D viscoplastic finite element model (FEM) for the FSW of thick aluminium profiles, in which the effect of the welding speed on the process was investigated. He found that the force acting on the tool increased with increasing travel speeds, while the opposite was observed at increasing rotational speeds. Good agreement between measured and predicted thermal fields was obtained, provided that the constitutive behaviour of the material for wide ranges of temperatures, strains, and strain rates being typical of FSW was properly accounted for. Moreover, Colegrove and Shercliff [17] have carried out a 2D numerical investigation of the effect of different pin geometries for FSW by employing the commercial CFD software package FLUENT. The material flow and forces arising during the process were investigated under both sticking and slip boundary conditions at the tool–matrix interface. They presented a novel “slip” model, where the tool–matrix interface condition was governed by the local shear stress. Furthermore, Schmidt and Hattel [18] have presented a fully coupled thermomechanical 3D finite element model based on the Lagrangian–Eulerian formulation and the Johnson–Cook constitutive material law and used it to analyse the governing mechanisms responsible for the filling of the cavity behind the tool. In their work, the contact forces were modelled according to Coulomb’s law of friction. In addition, the separation between tool and workpiece was allowed for in the model.

In addition, uncoupled thermomechanical numerical models exist in the scientific literature for FSW [20,21,22]. They are not as predictive as the coupled thermomechanical models but offer instead a more comprehensive understanding of the heat generation under different welding conditions. For instance, Chao et al. [20] measured the temperatures in both the tool and the workpiece during FSW and used the transient temperature fields to determine the heat flux generated by friction via finite element analysis. They found that most of the heat was transferred from the tool to the workpiece, while only a small fraction was absorbed by the tool itself. At the same time, they found that about 80% of the plastic work done within the thermomechanically affected zone was dissipated as heat.

Considerable effort has also been made to formulate analytical models for FSW. For example, an analytical model for the heat generation under a different tool–matrix interface interaction conditions was proposed by Schmidt et al. [23]. Based on a comparison between model predictions and experiments, they concluded that the tool–matrix interaction was characterised by sticking or near-sticking friction conditions.

However, due the novelty and uniqueness of the HYB process involving the use of FM additions, all these models previously developed for FSW are not readily transferable but need to be modified to allow for the flow of the FM through the extruder and into the groove. In a previous study by the authors, a finite element (FE) thermal model for the HYB process has been developed by exploiting the framework provided by the numerical code WELDSIM [24]. The model allows both the thermal and microstructure fields along with the resulting HAZ hardness profile to be calculated from knowledge of the net power input. So far, the FE model has been applied to butt welding of 4 mm aluminium profiles of the AA6082-T6 type using a ϕ1.4 mm filler wire (FW) of matching composition. Based on a best-fit comparison between predicted and measured thermal cycles for two different positions within the HAZ, a thermal efficiency factor of 0.28 was obtained for the HYB PinPoint extruder [24]. This means that only a minor fraction of the heat being generated, as calculated from the torque acting on the rotating drive spindle, is actually absorbed by the base plates in a real joining situation.

In the present investigation, a simpler and much faster method for calculating the net heat generation during HYB is presented, building on what have been done previously for friction stir welding (FSW) [25]. In that FSW study, the classical Rosenthal thin plate solution for the pseudo-steady state temperature distribution around a fully penetrating line source is used as a starting point for the calculations. Then, the resulting heat generation is computed from a separate frictional heating model, which considers the individual contributions from the rotating pin and tool shoulder on the net power input. When the two models are coupled, the solution algorithm converges after a few iterations and yields thermal fields that are in close agreement with those inferred from in situ thermocouple measurements [25]. Moreover, because the FSW heat generation model is physically based, it also exhibits an inherent predictive power. This makes the approach particularly attractive in the HYB case for reasons that will be obvious from the arguments provided below.

## 2. How the HYB Process Control System Works

In the HYB process, the control of the PinPoint drive spindle rotational speed is managed by an electric motor that is provided with a fixed gearing. During extrusion and joining, the torque acting on the drive spindle may vary, depending on the experimental conditions. Still, the HYB control system guarantees a constant spindle rotational speed [14]. Hence, the spindle rotational speed, as opposed to the torque, is a process variable that can be fixed prior to the welding operation and used to control the FM addition.

However, as already pointed out in the introduction, only a minor fraction of the total heat being generated in the HYB process is actually absorbed by the underlying base plates in a real joining situation. As a matter of fact, most of the power output is consumed in redundant frictional work between accumulated aluminium flash and the rotating pin, the abutment, and the slide bearing inside the stationary housing. Therefore, the drive spindle torque has no real physical meaning in the sense that it provides a measure of the plastic deformation work imposed on the FM and the BM in the groove. Still, based on $M_{t}$ (Nm) and the applied drive spindle rotational speed $N_{s}$ (RPM), it is possible to calculate the total power consumption during extrusion and joining $W_{t}$(W) from the relationship [10]:

(1) $$W_{t}=\;\frac{2\;\pi }{60}\;M_{t}\;N_{s}.$$

Then, after adjusting for the amount of heat per unit time $W_{c}$(W) being simultaneously removed from the extruder by the CO_2_ coolant used to control temperature in the FW inlet hole in the stationary housing, we obtain the gross power input $W_{g}$ (W) during extrusion and joining:

(2) $$W_{g}=\;W_{t}-\;W_{c}.$$

Since $W_{g}$ is analogous to the total power input in conventional electric arc welding, it can be used to calculate the gross heat input E (in kJ/mm) for the HYB process when the extruder travel speed v (mm/s) is fixed [10]:

(3) $$E=\;\frac{W_{g}}{1000\;\;v}.$$

This parameter is widely used for specifying the heat input in ordinary fusion welding and is therefore equally applicable in the HYB case for documenting process control. However, because the parameter E is calculated on the basis of the gross power input during welding and not the net power input, it is not possible to compare these values with those of other welding processes [4]. Therefore, the development of a more physically-based model for the heat generation and the net power input in HYB is highly needed, particularly if it also displays a certain degree of predictive power. This will enable better process control and optimisation of weld properties through modelling and at the same time allow benchmarking against other commercial welding methods under comparable experimental conditions.

## 3. Components of the HYB Heat Generation Model

The HYB heat generation model consists of a heat flow model being coupled to a frictional heating model.

### 3.1. Heat Flow Model

**Figure 2:**
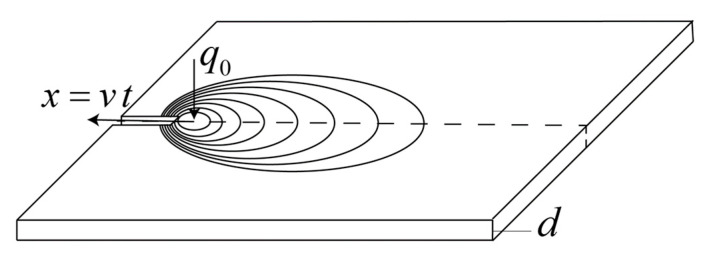
Outline of the analytical 2D heat flow model for HYB butt welding.

In single-pass aluminium butt welding, the classical Rosenthal thin-plate solution can be employed for prediction of the temperature distribution at pseudo-steady state [4]. Provided that the net power input $q_{0}$ (W), the plate thickness *d* (mm), and the extruder travel speed *v* (mm/s) are kept within the range normally applicable to thin sheet aluminium welding, the isotherms close to centre-line will approximately be circular in shape also in the HYB case, as shown schematically in Figure 2. At distances further away, they tend to become increasingly elongated.

Under 2D heat flow conditions, the pseudo-steady state temperature distribution around the fully penetrating line source is given by the following equation [4,26,27]:

(4) $$T=T_{0}+\frac{q_{0}/d}{2\;\pi \;\lambda }\;\exp\left[-\frac{v\;x}{2\;a}\right]K_{0}\left(\frac{v\;r}{2\;a}\right)$$

where $T_{0}$ (°C) is the initial (preheating) temperature of the aluminium plates, r (mm) is the radius vector referred to the centre of the penetrating line source, and $K_{0}$ is the modified Bessel function of second kind and zero order. The other symbols have their usual meaning and are defined in Appendix A.

It is noted that the Rosenthal thin plate solution omits a consideration of heat conduction occurring in the through-thickness direction of the weld and further into the underlying steel backing plate. In addition, boundary effects are neglected. This means that it is only applicable under conditions where the assumption of 2D heat flow is reasonable.

### 3.2. Frictional Heating Model

**Figure 3:**
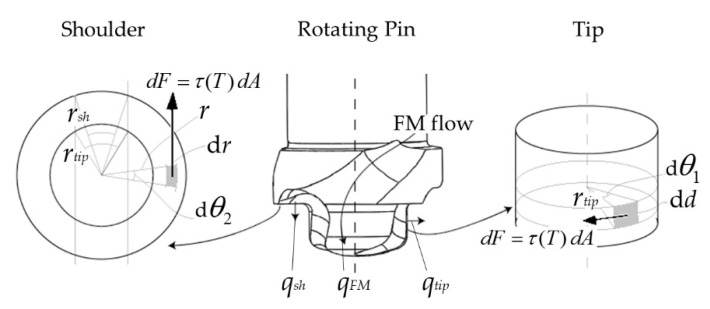
Schematic drawings of the rotating pin with its protruding cylindrical tip and flat shoulder. As an aid to the reader, some of the symbols and parameters used in deriving the mathematical expressions for the net heat generation are also highlighted.

Figure 3 shows contour drawings of the rotating pin with its protruding cylindrical tip and flat shoulder, into which the helicoid-shaped moving dies for the FM flow are imprinted. From this figure, it is evident that the net heat generation $q_{0}$ (W) in the HYB case due to interactions between the rotating pin and the underlying base plates can be written as the sum of the following three contributions:

(5) $$q_{0}=q_{tip}+\;q_{sh}+\;q_{FM}$$

where $q_{tip}$, $q_{sh}$, and $q_{FM}$ refer to the individual contribution from the tip, the shoulder, and the FM, respectively, as defined in Figure 3.

In friction-driven processes such as HYB and FSW, the contact conditions between the rotating pin and the underlying base plates will determine the extent of heat being generated during welding [25]. According to Schmidt et al. [23,28], there are three kinds of contact states that are relevant under the prevailing circumstances; sticking, sliding, or a combination of both (mixed state). When aluminium sticks to the tool surface, the heat generation will be controlled by the local aluminium yield strength in shear $\tau_{yield}$ (MPa) because the tool then is allowed to move relative to the underlying aluminium through sub-surface shearing. Similarly, under sliding conditions, all relative movement between the tool and the aluminium occurs at the contact surface. In that case, the heat generation is determined by the friction shear stress $\tau_{friction}$ (MPa).

The mixed state is more difficult to handle. However, if one assumes, as a first approximation, that $\tau_{yield}\;\approx \;\tau_{friction}=\;\tau$ at the same time as the strain rate dependence of the yield stress is neglected, the task becomes manageable [25]. Note that the latter assumption is not unrealistic in the HYB case because the angular velocity ω (rad/s) of the rotating pin is always kept constant for a given welding operation. In addition, this is not a parameter that varies significantly from one weld to another [10,11].

**Figure 4:**
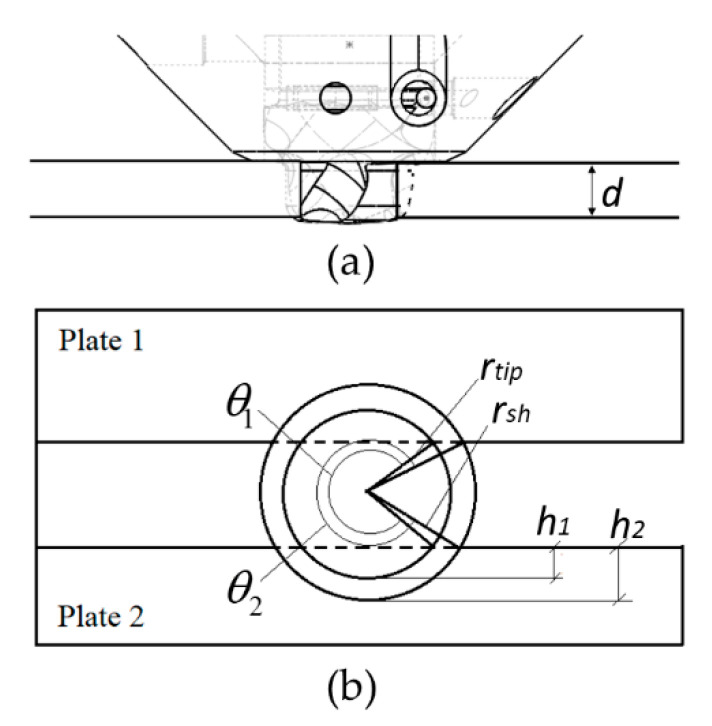
Schematic drawings defining the contact conditions during butt welding of aluminium plates using the HYB PinPoint extruder; (**a**) “Pin-in-groove” situation, (**b**) “Bird-eye” view of the rotating pin inside the groove.

The contact conditions existing during butt welding of two aluminium plates of thickness d (mm) using the PinPoint extruder are further highlighted in Figure 4. In the present experimental setup, the tip is positioned symmetrically inside an open I-groove of width k (mm), which confines the contact area. This must be accounted for in the heat flux balance. Then, following the treatment of Ferro and Bonollo [25], recalling that τ (MPa) is a temperature-dependent parameter, the appropriate expression for $q_{tip}$ (W) in the HYB case becomes:

(6) $$q_{tip}=\omega \;\int_{A}\tau (T)\;r_{tip}\;dA=\omega \;\tau (T)\;r_{tip}{}^{2}\;\theta_{1}\;d\;.\;$$

In Equation (6) $\theta_{1}$ (rad) is a geometrical term defining the contact area between the tip and the aluminium groove walls, which for the specific groove geometry shown in Figure 4 is given as:

(7) $$\theta_{1}=2\;\left[\pi -\arcsin\left(\frac{r_{tip}-h_{1}}{r_{tip}}\right)\right]$$

where $h_{\;1}$ (mm) is the difference between the half width k/2 (mm) of the groove and the tip radius $r_{\;tip}$ (mm).

The next step is to allow for the temperature dependence of the local yield shear stress τ(T). According to Ferro and Bonollo [25], a useful expression for τ(T) in the present context would be:

(8) $$\tau (T)=\tau_{{}_{yield}}^{0}\left(1-\frac{T_{\mathrm{int}}}{T_{eut}}\right)$$

where $\tau_{yield}^{0}$ (MPa) is the upper threshold value for the local yield shear stress, $T_{\mathrm{int}}$ (°C) is the aluminium–tool interface temperature, while $T_{eut}$ (°C) is the eutectic temperature of the aluminium alloy (i.e., the lower temperature at which local melting occurs). Note that when Equation (8) is used to obtain the interface temperature in the HYB case, it is implicitly assumed that $T_{\mathrm{int}}$ is constant around the entire pin surface periphery and equal to that calculated at a given distance r= r* from the weld centre-line. Therefore, r* (mm) can also be regarded as a fitting parameter in the heat generation model. For butt welding of aluminium plates using the HYB PinPoint extruder, the best overall agreement between predicted and measured thermal fields is obtained when $r*\approx \;r_{sh}$.

Similarly, the contribution from the pin shoulder can be expressed as:

(9) $$q_{sh}=\omega \;\int_{A}\tau (T)\;r\;dA\;=\omega \;\;\tau (T)\;(r_{sh}{}^{3}-r_{tip}^{3})\;\theta_{2}/3.$$

In Equation (9) $\theta_{2}$ (rad) is a geometrical term defining the contact area between the pin shoulder and the underlying aluminium base plates, which for the specific groove geometry shown in Figure 4 is given as:

(10) $$\theta_{2}=2\left[\pi -\arcsin\left(\frac{r_{sh}-h_{2}}{r_{sh}}\right)\right]$$

where $r_{sh}$ (mm) is the radius of the shoulder and $h_{2}$ (mm) is the difference between the half width k/2 of the groove and the shoulder radius $r_{sh}$ (mm).

Finally, it is necessary to include the contribution from the FM additions in the heat flux balance, as determined by the mass flow of aluminium through the extruder $\overset{\cdot }{m}$ (kg/s), the specific heat capacity of the filler metal $C_{p}$ (J kg^−1^ °C^−1^), and the temperature $T_{FM}$ (°C) of the FM at the time it leaves the extrusion chamber and starts to flow through the moving dies in the rotating pin and into the groove. In the HYB case, the appropriate expression for $q_{FM}$ (W) is:

(11) $$q_{FM}=\overset{\cdot }{m}\;C_{p}\;T_{FM}.$$

Then, by combining Equations (6)–(11), we arrive at the following expression for the net heat generation $q_{0}$ (W) in the HYB case due to interactions between the rotating pin and the underlying base plates:

(12) $$q_{0}=\omega \;\tau (T)r_{tip}{}^{2}d\;2\;\left[\pi -\arcsin\left(\frac{r_{tip}-h_{1}}{r_{tip}}\right)\right]+\;\omega \;\tau (T)\;(r_{sh}{}^{3}-r_{tip}{}^{3})\;\frac{2}{3}\;\left[\pi -\arcsin\left(\frac{r_{sh}-h_{2}}{r_{sh}}\right)\right]+\overset{\cdot }{m}C_{p}T_{FM}.$$

### 3.3. Coupling of Models

**Figure 5:**
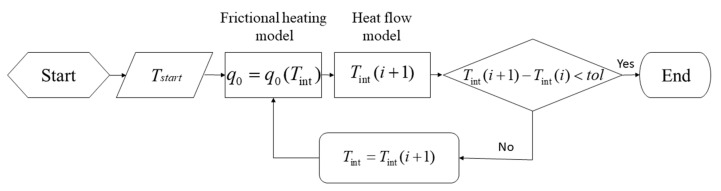
Flowchart showing the steps involved in calculating the interface temperature at position $r*=r_{sh}$ using the coupled heat flow and frictional heating models.

The prediction of the net power input and the resulting thermal fields being induced in the underlying aluminium base plates by the process is made by means of the Matlab iterative algorithm shown in Figure 5 that couples the frictional heating model to the heat flow model.

The algorithm presents the two main parts that interact with each other until convergence is achieved. In the first iteration, the frictional heating model calculates the initial value for $q_{0}$ via Equation (12), which is based on the chosen start-up temperature $T_{start}$. This equation takes into consideration both the pin geometry and the material properties at the tool–matrix interface. The calculated value is inserted back into the classical Rosenthal thin plate solution (Equation (4)) to obtain a new updated value for the interface temperature $T_{\mathrm{int}}$ at the same position, which then is used as the basis for calculating the next estimate of $q_{0}$ from Equation (12). The process is repeated until the difference $T_{\mathrm{int}}(i+1)-\;T_{\mathrm{int}}(i)$ is less than the chosen tolerance value and convergence is obtained. Usually, the solution algorithm converges after a few iterations, which makes the model quick and easy to implement and use in practice. 

Table 1 summarises the input data used in the simulations.

## 4. Materials and Experimental Conditions

The experimental validation of the semi-analytical heat generation model was done on the basis of special designed welding trials carried out under controlled laboratory conditions, using the pilot HYB machine (HyBond AS, NAPIC, Richard Birkelands vei 2b, 7491 Trondheim, Norway) at the Norwegian University of Science and Technology (NTNU). This machine allows welds to be produced under controlled conditions, with full documentation of all relevant process parameters, e.g., temperature, torque, rotational speed, travel speed, and wire feed rate as well as the main reaction forces acting on the extruder during welding [14].

The four millimetre thick AA6082 plates employed in the welding trials were obtained from a commercial aluminium manufacturer (ThyssenKrupp Materials Logist. Heinrich-August-Schulte-Str. 6, 44147 Dortmund, Deutschland). They were produced by hot rolling and received in the T6 temper condition. As a starting point, two test welds were first made using a fixed groove width of 3 mm and the welding parameters listed in Table 2. Note that these welding parameters, which are based on experience data, provide a sensible choice among several different alternatives to achieve adequate groove filling and good bonding conditions under the prevailing circumstances. Then, the two test welds were sectioned and examined metallographically to determine the exact position of the outer border area of the extrusion zone (EZ) on the advancing side (AS) of the welds. This examination revealed that the closest position inside the plates in which the thermocouples could be safely located was 3 mm, which referred to the groove wall surface, without risking having them destroyed during the welding operation due to the rotating action of the pin.

**Figure 6:**
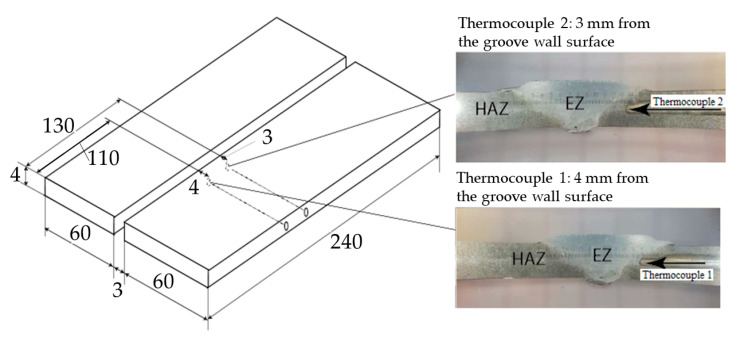
Workpiece dimensions and locations of thermocouples.

Then, two new sets of base plates were prepared. The plates located on the AS were both provided with guiding holes for the insertion of Φ1 mm thermocouples before the welding experiments were repeated again under identical operational conditions. In these experimental welds, the first thermocouple was placed 110 mm from the start position of the weld 4 mm from the groove wall surface, while the other one was placed 130 mm from the start position of the weld 3 mm from the groove wall surface, as shown in Figure 6. Copper paste was used to improve the contact between the thermocouples and the aluminum. The temperature–time histories were recorded using a digital data logger with a total capacity of four type-K (chromel-alumel) thermocouples. The applied sampling interval varied from 1 to 0.1 s, depending on the circumstances. This experimental setup allowed the pseudo-steady state thermal cycle during welding to be measured with a reasonable degree of accuracy at both thermocouple locations and compared with that calculated from the classical Rosenthal thin-plate solution (Equation (4)).

## 5. Results and Discussion

**Figure 7:**
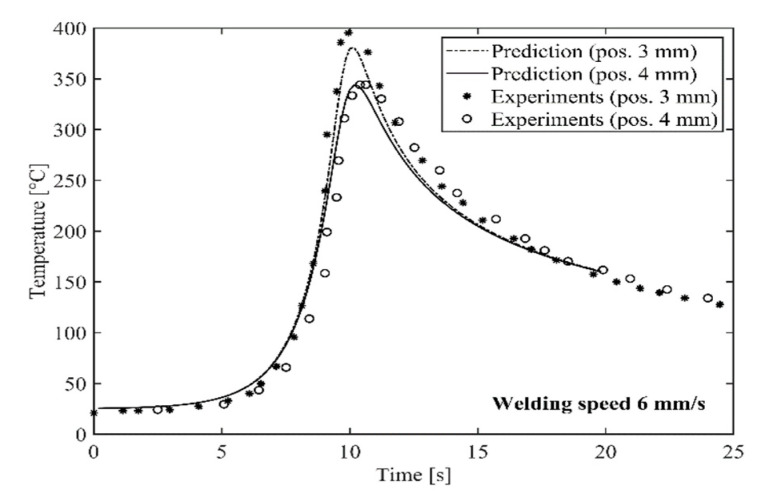
Comparison between predicted and measured thermal cycles for the butt weld produced at a welding speed of 6 mm/s. The indicated thermocouple locations are defined in Figure 6.

**Figure 8:**
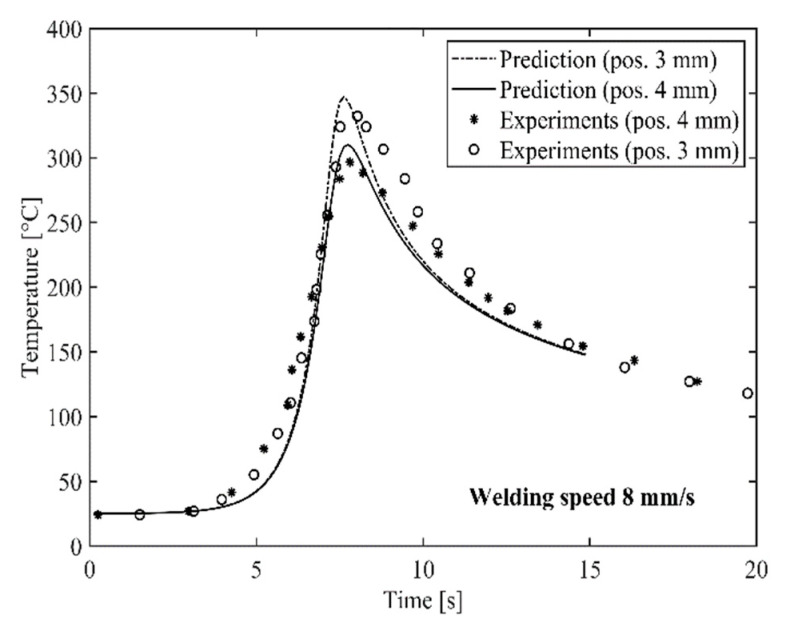
Comparison between predicted and measured thermal cycles for the butt weld produced at a welding speed of 8 mm/s. The indicated thermocouple locations are defined in Figure 6.

Figure 7 and Figure 8 show a comparison between predicted and measured thermal cycles for the two HYB butt welds produced at a welding speed of 6 and 8 mm/s, respectively. In both cases, it is evident that the combined heat flow and frictional heating models adequately reproduce the actual temperature–time programme at the indicated thermocouple locations. As shown in Table 3, the predicted net power input is 823 and 844 W, respectively. This means that the thermal efficiency factor η varies in the range from 0.26 to 0.27, as evaluated from a comparison with the listed value for the gross power output in Table 2.

It is noted that the computed η-values are in close agreement with those previously inferred from more sophisticated heat flow calculations using the numerical code WELDSIM [24]. Therefore, the present model predictions, which are surprisingly accurate, confirm previous findings that only a minor fraction of the heat being generated is actually absorbed by the underlying base plates in a real joining situation. This is true, although the Rosenthal thin plate solution omits a consideration of heat conduction in the through-thickness direction of the weld and further into the underlying steel backing plate.

**Figure 9:**
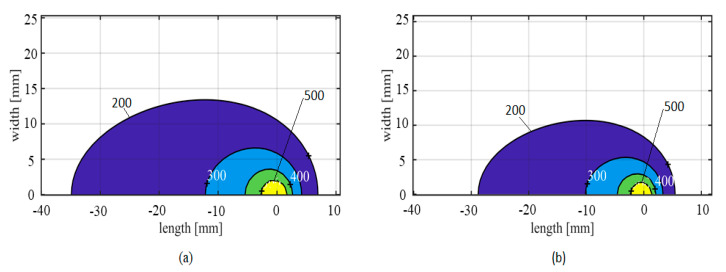
Calculated isothermal contour plots (degree Celsius) at pseudo-steady state for the same two butt welds referred to in Table 3; (**a**) Welding speed of 6 mm/s; (**b**) Welding speed of 8 mm/s.

Moreover, Figure 9 shows calculated contour plots of the isotherms for the same two butt welds, using input data from Table 3. It follows that the isotherms become increasingly elongated as the welding speed increases. This change of shape is to be expected, because it provides a manifestation of how fast the extruder head is moving compared to the rate at which the absorbed heat is removed by conduction in the base plates [4].

**Figure 10:**
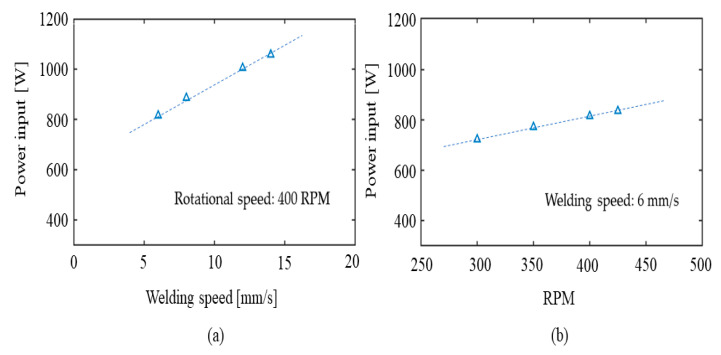
Sensitive analyses showing how the net power input responds to variations in the operational conditions; (**a**) Effect of welding speed on $q_{0}$ at a constant pin rotational speed of 400 RPM, (**b**) Effect of the pin rotational speed on $q_{0}$ at a constant welding speed of 6 mm/s.

It is also interesting to observe that the calculated value for the net power input is seen to increase with increasing welding speeds (see Table 3). This predicted behaviour is also physically reasonable in view of how the HYB process control system actually works. For example, if the increase in the welding speed is not compensated for by a corresponding increase in the pin rotational speed, one would intuitively expect that a higher spindle torque is needed to plastically deform the colder BM material being in contact with the rotating pin. In turn, this will raise the total power consumption and thus the net power input and trigger a response similar to that predicted in Figure 10a.

A more expected response is observed if the rotational speed is allowed to increase at the same time as the welding speed is kept constant, as illustrated Figure 10b. Again, the predicted behaviour is physically reasonable and consistent with how the HYB process control system actually works. It is also noted that similar results have been documented by other researchers [30,31] that developed analytical and FE thermal models for FSW and investigated the variation of the calculated and measured temperatures for different welding speeds. Other numerical models developed for FSW [31,32,33] showed the dependence of the thermal field arising for different rotational speed that agree with the trend found in the present investigation. For example, Jacquin et al. [31] plotted the predicted and measured evolution of the power consumed in the weld as a function of the welding parameters and showed the repartition of the predicted power dissipation as a function of the welding velocity, finding a correlation similar to the one presented in Figure 10. They also observed that although the total power generated in the weld increases with the welding velocity, the maximum temperature value decreases, and this is also in agreement with what was found in the present investigation of HYB.

Another interesting observation is that the predictions appear to be rather insensitive to changes in the input data, e.g., the chosen value for the adjustable $\tau_{yield}^{0}$ fitting parameter in the expression for the friction coefficient τ(Equation (8)) and the assumed dimensions of the rotating pin. This follows from an inspection of the supplementary modelling results presented in Appendix B (see Table A2 and Table A3, respectively). Therefore, the present heat generation model is deemed to exhibit the required degree of predictive power to make it useful in combination with more advanced numerical models for simulation of the thermal, microstructure and residual stress fields, where the thermal efficiency factor is an essential input parameter [24].

## 6. Conclusions

Since both HYB and FSW are friction-driven processes, the heat generation problem in a butt welding situation can be treated in a similar manner using the same physical framework. However, in the HYB case, it is also necessary to allow for the aluminium filler metal additions and the fact that welding always occurs inside a groove with a fixed spacing. This confines the contact area between the rotating pin and the aluminium base plates.

The HYB heat generation model developed for the butt welding of aluminium plates and profiles consists of a heat flow model and a frictional heating model that are coupled. As a starting point, the classical Rosenthal thin plate solution for the pseudo-steady-state temperature distribution around a fully penetrating line source is invoked. Then, the associated heat generation is calculated by considering the individual contributions from the tip of the rotating pin, the pin shoulder, and the filler metal additions on the net power input.

When the analytical heat flow model and the frictional heating model are coupled, the solution algorithm converges after a few iterations and yields thermal efficiency factors that are in close agreement with those obtained from more sophisticated finite element analyses. Hence, the model captures the heat generation in the HYB process surprisingly well with a minimum of computational effort. This is true, although the Rosenthal thin plate solution omits a consideration of heat conduction in the through-thickness direction of the weld and further into the underlying steel backing plate.

In the specific case where the HYB heat generation model is applied to butt welding of 4 mm aluminium plates of the AA6082-T6 type using a Φ1.4 mm filler wire of matching composition, a thermal efficiency factor of 0.27 is obtained for the HYB PinPoint extruder. This confirms previous findings that only a minor fraction of the heat being generated, as calculated from the torque acting on the rotating drive spindle, is actually absorbed by the base plates in a real joining situation.

Since the HYB heat generation model is physically based, it also exhibits an inherent predictive power. This makes it particularly useful in combination with more advanced numerical models for simulation of the thermal, microstructure, and residual stress fields, where the thermal efficiency factor is an essential input parameter.

## Figures and Tables

**Table 1:** Summary of input data used in the semi-analytical model.

Symbol	Value	Units	Source
ρ	2700	kg/m^3^	[4]
$C_{p}$	896	J/(kg°C)	[4]
λ	167	W/(m°C)	[4]
α	$62\times \;10^{-6}$	m^2^/s	[4]
r*	$5.5\times \;10^{-3}$	m	-
$T_{0}$	25	°C	-
$\tau_{yield}^{0}$	$69\;\times 10^{6}$	Pa	[29]
ω	42	rad/s	-
$r_{tip}$	$2.75\times \;10^{-3}$	m	-
d	$4\;\times 10^{-3}$	m	-
$r_{sh}$	$5.5\times \;10^{-3}$	m	-
$T_{eut}$	583	°C	[4]
$\overset{\cdot }{m}$	$4.33\;\times 10^{-4}$	kg/s	-
$T_{FM}$	450	°C	[14]

**Table 2:** Summary of operational conditions used in the two HYB butt welding trials.

I-Groove Width	Welding Speed	Pin Rotational Speed	Wire Feed Rate	Gross Power Output ^†^
3 mm	6 and 8 mm/s	400 RPM	142 mm/s	3135 W

^†^ Value is not corrected for the amount of heat per second being removed by the CO_2_ gas used to cool down the extruder.

**Table 3:** Predicted values for the net power input during butt welding 4 mm thick plates of AA6082-T6 at two different welding speeds using the HYB PinPoint extruder.

Welding Speed	Pin Rotational Speed	Net Power Input	Thermal Efficiency Factor
6 mm/s	400 RPM	823 W	0.26
8 mm/s	400 RPM	844 W	0.27

## Data Availability

Data is contained within the article.

## Appendix A

**Table A1:** Nomenclature and symbols.

Symbol	Description
a	Thermal Diffusivity, m^2^/s
A	surface area, m^2^
$C_{p}$	specific heat capacity, J/(kg °C)
d	plate thickness, m
E	gross heat input during extrusion and joining, J/m
$h_{1}$	distance, as defined in Figure 4, m
$h_{2}$	distance, as defined in Figure 4, m
k	groove width, m
$K_{0}$	Bessel function of the second kind and zero order
$\overset{\cdot }{m}$	filler metal flow rate, kg/s
$M_{t}$	torque acting on drive spindle, Nm
$N_{s}$	spindle rotational speed, RPM
$q_{0}$	net power input due to frictional heating and plastic deformation, W
$q_{FM}$	contribution from to the filler metal additions to the net heat generation, W
$q_{sh}$	contribution from the rotating pin shoulder to the net heat generation, W
$q_{tip}$	contribution from the rotating pin tip to the net heat generation, W
r	radial distance from centre of heat source, m552
$r_{sh}$	pin shoulder radius, m
$r_{tip}$	tip radius, m
$r^{*}$	fixed value for *r* in heat generation model, m
*t*	time, s
T	temperature, °C
$T_{0}$	initial temperature, °C
$T_{eut}$	eutectic temperature of alloy, °C
$T_{FM}$	filler metal temperature, °C
$T_{\mathrm{int}}$	aluminium–tool interface temperature, °C
v	welding speed, m/s
$W_{c}$	heat per unit time removed from the extruder by the CO_2_-coolant, W
$W_{g}$	gross power input during extrusion and joining, W
$W_{t}$	total power consumption during extrusion and joining, W
x	welding direction, m
λ	thermal conductivity, W/(m °C)
ρ	density, kg/m^3^
η	thermal efficiency factor
ω	angular rotation speed, rad/s
τ	local yield shear stress, Pa
$\tau_{friction}$	friction shear stress, Pa
$\tau_{yield}$	local aluminium yield strength in shear, Pa
$\tau_{yield}^{0}$	upper threshold value for τ, Pa
$\theta_{1}$	correction term, as calculated from Equation (7), rad
$\theta_{2}$	correction term, as calculated from Equation (10), rad

## Appendix B

**Table A2:** Analysis of the sensitivity of the model outputs to variations in the $\tau_{yield}^{0}$ fitting parameter.

$\tau_{yield}^{0}$ (MPa)	$T_{peak}$ (° C), pos. 3 mm		Err%	$T_{peak}$ (° C), pos. 4 mm		Err%
	Predictions	Experiments		Predictions	Experiments	
69	380.9	395.4	−3.7	343.8	344,2	−0.12
64	367.3	395.4	−7.1	332	344.2	−3.54
58	352	395.4	−11.0	319	344.2	−7.32
75	390.1	395.4	−1.3	353.1	344.2	2.59

**Table A3:** Analysis of the sensitivity of the model outputs to variations in the pin dimensions.

$r_{tip}$ (mm)	$r_{sh}$ (mm)	$T_{peak}$ (° C) pos. 3 mm	$T_{peak}$ (° C) pos. 4 mm
2.75	5.5	380.9	343.8
2.5	5.5	374.4	337.3
2.75	5.25	366.5	330.9
3	5.5	388.1	351.4
2.75	5.75	391.9	353.7
2.5	5.25	358.9	324.1
